# Retraction Note to: Targeting MALAT1 and miRNA-181a-5p for the intervention of acute lung injury/acute respiratory distress syndrome

**DOI:** 10.1186/s12931-022-02066-x

**Published:** 2022-06-25

**Authors:** Yaling Liu, Xiaodong Wang, Peiying Li, Yanhua Zhao, Liqun Yang, Weifeng Yu, Hong Xie

**Affiliations:** 1 Department of Anesthesiology, The Second Afliated Hospital of Soochow University, 1055 Sanxiang Road, Suzhou, 215004 Jiangsu China; 2 Department of Anesthesiology, Renji Hospital, Shanghai Jiaotong University School of Medicine, 160 Pujian Road, Shanghai, 200127 China; 3 Department of Cardiology, Shanghai East Hospital, Tongji University School of Medicine, Shanghai, China

## Retraction to: Respir Res (2021) 22:1 10.1186/s12931-020-01578-8

The Editor-in-Chief has retracted this Article. After publication, concerns were raised regarding the methodology and data presented, specifically:

- The source of primary HPMECs is incorrectly stated as ATCC, and the cell culture conditions described in the Methods are unsuitable for primary cells.
- The flow cytometry assay described in the Methods does not match the presented data.
- The same western blot control bands are used in Figs. 2 and 3, and in Figs. 4 and 5.

The authors have provided raw flow cytometry data, which is inconsistent with the results presented in the Article and contains duplication between various samples. The authors have been unable to provide full uncropped western blot gel images for verification upon the Editors' request. Additionally, the Article was simultaneously submitted to and published by another journal [1, retracted]. The Editor-in-Chief therefore no longer has confidence in the presented data and the originality of this work.

All authors agree to this retraction.
